# Chitosan and Sodium Alginate Implementation as Pharmaceutical Excipients in Multiple-Unit Particulate Systems

**DOI:** 10.3390/polym14142822

**Published:** 2022-07-11

**Authors:** Martina Čierna, Pavel Mučaji, Miroslava Špaglová, Mária Čuchorová, Oliver Macho

**Affiliations:** 1 Department of Galenic Pharmacy, Faculty of Pharmacy, Comenius University Bratislava, 832 32 Bratislava, Slovakia; spaglova@fpharm.uniba.sk (M.Š.); cuchorova@fpharm.uniba.sk (M.Č.); 2 Department of Pharmacognosy and Botany, Faculty of Pharmacy, Comenius University Bratislava, 832 32 Bratislava, Slovakia; mucaji@fpharm.uniba.sk; 3 Institute of Process Enginering, Faculty of Mechanical Engineering, Slovak University of Technology in Bratislava, 812 31 Bratislava, Slovakia; oliver.macho@stuba.sk

**Keywords:** chitosan, sodium alginate, pellets, multi-unit pellet system, hydroxypropyl methylcellulose, acyclovir

## Abstract

This study aimed to prepare and evaluate pellets containing acyclovir as a model drug. Pellets were prepared by the extrusion–spheronization process. Aqueous solutions of natural marine polymers (sodium alginate, chitosan) were compared to semi-synthetic hydroxypropyl methylcellulose (HPMC) in the role of binders. The study focused on the characterization of the pellet properties that are crucial for the formulation of the final dosage form, such as in multi-unit pellet system (MUPS) tablets or hard gelatin capsules filled with the pellets. Finally, the mentioned dosage forms were tested for drug dissolution. The morphology of pellets observed by scanning electron microscopy correlated with the shape evaluation performed by dynamic image analysis. Sodium alginate pellets exhibited the lowest value of sphericity (0.93), and many elongated rods and dumbbells were observed in this batch. Chitosan pellets had the highest value of sphericity (0.97) and were also less rough on the surface. The pellets maintained a constant surface geometry during the dissolution studies; they only reduced in size. The most significant reduction in size and weight was assessed after 2 h of dissolution testing. This fact was in line with the drug release from pellets in capsules or MUPS tablets, which was massive during the first hour, in both cases. The dissolution profiles of all of the batches were comparable.

## 1. Introduction

The interest in the application of chitosan in industry, including in pharmaceuticals, is growing. The suitability and performance of chitosan as a component of formulations for drug delivery applications has been investigated in numerous studies. These include controlled drug delivery applications, use as a component of mucoadhesive dosage forms [1,2], rapid release dosage forms [3,4,5], improved peptide delivery [6,7], colonic drug delivery systems [8], and use for gene delivery [9]. The use of biocomposites based on chitosan in the field of tissue engineering is being widely studied. In the processes of tissue healing, chitosan serves as an extracellular matrix for cellular organization, helping in the attachment, proliferation, and differentiation of living cells [10]. Chitosan can also be used as a unique eco-friendly active bio-sorbent for the sorption of heavy metal ions from polluted water. It is commonly extracted from marine waste such as shrimp shells or crustacean waste [11]. Chemically speaking, it is a polysaccharide comprising copolymers of glucosamine and N-acetylglucosamine [12]. Whereas chitosan is soluble in acidic media, it shows no solubility in neutral and alkaline aqueous solutions. This is in contrast to a lot of other high-molecular-weight polymers that show a neutral or anionic character [13]. The polycationic nature of chitosan makes it a bioadhesive that readily binds to negatively charged surfaces such as mucosal membranes. Thereby it increases the adhesion to the mucosa and, as a result, enhances the time of contact for penetration of drug molecules. Hence, chitosan has an absorption-promoting effect due to its mucoadhesion properties, but also due to transient opening of the tight junctions of the mucosal cell membrane [14]. 

Alginates have been extensively studied for oral, parenteral, pulmonary, and transdermal drug delivery [15]. They are mainly derived from brown algae, but can also be extracted from bacterial sources [16]. Sodium alginate consists of the sodium salt of alginic acid, which is a mixture of polyuronic acids composed of residues of D-mannuronic acid and L-guluronic acid [17,18]. Sodium alginate can be used alone or in the presence of calcium ions, because one of the most important and useful properties of alginates is its ability to form gels in the presence of multivalent metal ions, such as calcium. The incorporation of calcium salts in pellet formulations altered the drug release, depending on the solubility of the calcium salts used. However, in most cases, it was only a slightly slower drug release [15]. As the use of calcium ions did not have a significant influence on the drug release from pellets made by the extrusion–spheronization process, it was decided to prepare alginate pellets without calcium ions. 

Methocel K100M (hydroxypropyl methylcellulose, HPMC, hypromellose) was used as a reference polymer, with the intention of comparing the physicochemical properties and dissolution behavior of the pellets containing natural polymers and pellets containing well-established cellulose ether. Hypromellose hydrophilic matrix systems have been widely studied and many successful products on the market utilize this versatile extended release technology [19]. Hypromellose is an excellent excipient for formulation of classical dosage forms and advanced drug delivery systems. New methods of hypromellose processing include spray draying, hot-melt extrusion, 3D printing, and electrospinning [20].

Pellets are described as geometrically-defined agglomerates, they are free-flowing, spherical or semi-spherical solid units with a size range of about 0.5–1.5 mm and are intended for oral administration [21]. They differ from granules and conventional agglomerates in shape, surface area, and narrow particle size distribution [22]. Pellets are used to control the drug release from the formulation or to prevent dose dumping. They offer reduced variation in gastric emptying rate and intestinal transit time, and also disperse freely in the gastrointestinal tract. Pelletization is a way to separate incompatible drugs or mask an unpleasant taste [21]. Pellets can also reduce irritant effects on the gastric mucosa [23]. Pharmaceutical pellets are typically manufactured via extrusion–spheronization. Afterwards, pellets are usually subjected to dissolution studies and screening, to achieve the desired physiochemical properties and size distribution; for which, we successfully applied dynamic image analysis (DIA) [21]. DIA is a modern particle characterization method with exclusive 3D measurement software. Compared with the other traditional particle evaluation techniques, DIA has the major advantage that the instrument provides images of fast moving particles and is sensitive to differences in size and shape characteristics; therefore, it is being increasingly applied to particle evaluation in various processes, pharmaceuticals included [24]. DIA has already been used in many studies for different types of materials: pharmaceutical excipients, minitablets, tailings, talc, concrete aggregates, sediments, volcanic ash, calcite, and coal [25]. Pellets, often also coated, are administered in the form of hard gelatin capsules or multi-unit pellet system (MUPS) tablets that quickly disperse in the stomach [26,27]. 

Acyclovir, the model drug used in this study, is the most widely used drug for infections of cutaneous herpes, genital herpes, chicken pox, varicella zoster infections, and herpes keratitis. Oral acyclovir is generally used five times a day (200 or 400 mg tablets) [28]. Frequent dosing of acyclovir is based on the physicochemical properties of acyclovir. Acyclovir is described as ‘‘slightly soluble in water’’ in different pharmacopoeias. The partition coefficient (log P) in n-octanol at 22 °C is 1.57. A log P value greater than that of metoprolol (1.72) indicates high permeability. As the log P value reported for acyclovir lies far below that value, it is expected to have low permeability. Acyclovir’s absolute bioavailability following oral administration has been reported to be in a range of 10–30% [29].

This study aimed to determine whether the natural polymers, sodium alginate and chitosan, are full-fledged substitutes for semi-synthetic HPMC in the role of a wetting agent in the formulation of pellets and to what extent they affect the quality properties of pellets, compressed MUPS tablets, as well as the in vitro dissolution of a model drug, acyclovir. 

Some research papers have focused on chitosan pellet production by the extrusion–spheronization process, but there is a lack of the papers which have also focused on the transformation process into the final MUPS tablet dosage form [13,30,31]. MUPS tablets are composed of tablets containing uncoated or coated pellets. The compaction process of pellets to form the MUPS tablet can cause variations of the mechanical properties and dissolution behavior of the drug. According to some studies, MUPS tablet formulations could enhance the membrane permeation of drugs, specifically by inclusion of functional excipients in MUPS tablet formulations. Interest has been shown in the use of mucoadhesive polymers as functional excipients for this purpose [32]. This might be a way to enhance the bioavailability of poorly bioavailable APIs such as acyclovir; therefore, we formulated pellets with a content of chitosan or sodium alginate.

## 2. Materials and Methods

Materials used for the pellet formulation were acyclovir of European Pharmacopea quality, which was obtained from Union Quimico Farmaceutica S.A., Barcelona, Spain. Chitosan (medium molecular weight 90–310 kDa, degree of deacetylation 82%) and sodium alginate (molecular weight 120,000–190,000 g/mol) were purchased from Sigma-Aldrich Chemie GmbH, Steinheim, Germany, Methocel K100M; co-processed microcrystalline cellulose with lactose monohydrate and natrium carboxymethyl cellulose (Specicell^®^140) were supplied by The Dow Chemical company, Michigan, USA. Acetic acid, sodium chloride, and hydrochloric acid were purchased from Centralchem s.r.o, Bratislava, Slovakia. Pellets were filled into hard gelatin capsules, which were provided by Interpharm a.s., Bratislava, Slovakia. Purified water was freshly prepared by distillation.

### 2.1. Preparation of Pellets

The binder solutions were prepared by dispersing polymers in purified water (chitosan 2%, *w*/*w*, sodium alginate 2%, *w*/*w*, hydroxypropyl methylcellulose 2%, *w*/*w*). Chitosan solution was acidified using acetic acid (3%, *w*/*w*). The powdered components, specifically acyclovir and co-processed microcrystalline cellulose with lactose monohydrate, were homogenized and subsequently wetted with the solutions of binders in a mixer–granulator Diosna planetary mixer P1-6 (Diosna GmbH, Osnabrück, Germany). The wet homogenized blend was extruded with a Gabler Laboratory extruder (Gabler Engineering GmbH, Malsch, Germany) through a shaping die of 0.8 mm diameter, while the speed of the rotating screw was 40 rpm. The extruded product was broken and spheronized with a Gabler Spheronizer (Gabler Engineering GmbH, Malsch, Germany), with the speed of the rotating plate 1200 rpm for 3 min. Pellets were dried in a fluid bed dryer Glatt GPCG2 Lab System (Glatt GmbH, Binzen, Germany). The temperature of the inlet air was 50 °C, and the drying process was maintained till the pellet moisture was less than 3%, measured using a Mettler-Toledo Halogen Moisture Analyzer HG63 (Mettler-Toledo GmbH, Greifensee, Switzerland). Three different batches of pellets were prepared, varying in the kind of binder used for preparation (Sodium alginate, chitosan, Methocel K100M hereinafter referred to as HPMC).

### 2.2. Characterisation of Pellets

#### 2.2.1. Surface Morphology

The morphology of the pellets was characterized in greater depth using a scanning electron microscope, Tescan Vega3 (Tescan Orsay Holding, a.s., Brno, Czech Republic). It was used for examination at magnifications of 45×, 150×, and 300×.

#### 2.2.2. Measurement of Pellet Size, Shape, and Sphericity

There are several methods for determining the shape and size of particles based on the principle of image analysis. One such method is dynamic image analysis (DIA), in which particles are captured in motion by digitalizing photos of each particle from a camera and storing them in an image file. The particles tumble and rotate, and are illuminated by stroboscopic light. The images are used to calculate morphological parameters based on the known size and location of the pixels in each image. DIA, unlike other techniques, can report different size parameters as it processes the image of particles. The size distribution and shape of the pellets were analyzed using a PartAn 3D device (Microtrac MRB, Haan, Germany). 

The characteristic dimension for determining the size of a pellet was the area equivalent diameter (*D*_a_) Equation (1), obtained from multiple photos of individual pellets.

(1) $$D_{a}=\sqrt{4A}/\pi$$

where *A* is the area of the projected particle. 

Mean pellet size (dmean) was calculated according to the relationship in Equation (2).

(2) $$d_{\mathrm{mean}}=\frac{\sum^{\;}\;\%\;in\;class\times mid.class\;size\;}{number\;of\;classes\;}/100$$

Various shape parameters such as sphericity (φ) were used to determine the shape of the pellets, Equation (3).

(3) $$\varphi =\frac{D_{a}}{D_{p}}$$

A sphericity value φ = 1 corresponds to a perfect sphere [25].

We also observed the mean pellet size change and sphericity change during the dissolution testing of the pellets, which were withdrawn from the dissolution apparatus after 2, 4, and 6 h. 

#### 2.2.3. Particle-Size Distribution Estimation by Analytical Sieving

A sieve analysis was performed on a Test Sieve Shaker Haver EML 200 digital T (Haver & Boecker, Oelde, Germany). Sieve sizes used for the analysis were 250 µm, 355 µm, 500 µm, 710 µm, 900 µm, and 1250 µm, and the time of agitation was 5 min. The weight of material retained on each sieve was accurately determined. This test gives the weight percentage of pellets in each sieve size range.

#### 2.2.4. Mechanical Resistance of Pellets

The pellets (15 g) were poured into a glass vial and put into a laboratory shaker to agitate for 10 min. Then the pellets were sieved through the sieve with a nominal aperture of 250 µm and weighed again. The pellet loss after agitation, expressed as a percentage mean of three parallel measurements, corresponds to the mechanical resistance. 

#### 2.2.5. Determination of Matrix Erosion

Determination of matrix erosion was performed in the same apparatus, under the same conditions, as the dissolution testing. The baskets containing 0.4 g of pellets were removed from the dissolution medium after 2, 4, and 6 h and dried to a constant weight at 50 °C. The difference between the initial and the final weight was expressed as the percentage of matrix erosion [30].

### 2.3. Resistance to Crushing of MUPS Tablets

The resistance to crushing of MUPS tablets was tested using a tablet hardness tester Schleuniger 2E (Dr. K. Schleuniger & Co., Solothurn, Switzerland). Ten tablets were placed into the apparatus horizontally, the force (N) needed for their crushing/destruction was recorded and expressed as a mean ± SD.

### 2.4. Dissolution Profiles

The pellets were filled into hard gelatin capsules or compressed into MUPS tablets. A rotary tablet press machine (Romaco Kilian, Cologne, Germany) was set up to produce tablets of average weight 0.300 g and a hardness between 50 to 80 N. Gelatin capsules were filled manually with 0.400 g of pellets. In vitro dissolution testing was performed using a basket-type dissolution tester Erweka DT 6 (Erweka GmbH, Langen, Germany). A simulated gastric fluid without pepsin, prepared by dissolving 2.0 g of sodium chloride in 7.0 mL of hydrochloric acid, replenished with purified water up to 1000 mL, was used as the dissolution medium (pH value approximately 1.2). The rotation of baskets was set to 50 rpm. The dissolution testing lasted 6 h, while the dissolution medium was permanently heated to 37 ± 0.5 °C. The samples of dissolution medium were withdrawn at fixed intervals (5, 10, 15, 30, 45, 60, 90, 120, 180, 240, 300, 360 min). The released amount of acyclovir in solutions was detected spectrophotometrically at 255 nm on a Genesys™ 10S UV-Visible Spectrophotometer (Thermo Scientific, Waltham, MA, USA) against a blank (the dissolution medium).

### 2.5. The Similarity of Dissolution Profiles

The dissolution profiles were compared through the determination of the difference factor *f*_1_ and similarity factor *f*_2_. The difference factor *f*_1_ expresses the percent difference between the two dissolution profiles at each time point. It is a measurement of the relative error between the two profiles [33]. It is calculated as a sum of the absolute values for the differences between the tested product (*T*) and the reference product (*R*), relative to the sum of the mean percentage of the released drug from the reference product, as Equation (4) describes:

(4) $$f_{1}=\left\{\frac{\left[\Sigma_{t=1}^{n}\;\left|R_{t}-T_{t}\right|\right]}{\left[\Sigma_{t=1}^{n}\;R_{t}\right]}\right\}\times 100\;$$

The similarity factor *f*_2_ is a logarithmic reciprocal square root transformation of the sum of squared differences between the profiles of the tested and the reference product. It represents the measurement of similarity in the percentage (%) of dissolution between the “average” dissolution profiles, where n is the number of time points and *R_t_* and *T_t_* are the mean percentages of the released drug from the (*R*) and (*T*) products [34]:

(5) $$f_{2}=50\times log\left\{{\left[1+\left(\frac{1}{n}\right)\sum_{t=1}^{n}{\left(R_{t}-T_{t}\right)}^{2}\right]}^{-0.5}\times 100\right\}\;$$

## 3. Results and Discussion

### 3.1. Preparation of Pellets

The studied formulations contained API acyclovir, which is a Class III drug according to the biopharmaceutics classification system (BCS), considering tablet strengths up to 400 mg. However, 800-mg acyclovir tablets belong to BCS Class IV [29].

As a filler, an excipient was used with the brand name Specicel^®^ 140. It is co-processed microcrystalline cellulose with lactose and sodium carboxymethyl cellulose. For many pharmaceutical applications, extruded pellets are achieved in suitable quality if a sufficient portion of microcrystalline cellulose is added. Microcrystalline cellulose (MCC) is very often also part of the pellet base, thanks to its ability to retain large amounts of water in its structure, which provides an elastoplastic wet mass suitable for successful extrusion and desired product quality. However, in some cases, incompatibilities of MCC have been reported because of its tendency to adsorb drugs on fibrils and cause possible chemical interactions [13,35]. For highly water-soluble drugs, a coating step is required to control the release of the drug. Alternatively, the release controlling polymer can be incorporated into the beads as a binder that can potentially control drug release, with or without a thin coating. These matrix pellet systems have advantages over coated pellets, due to their easier production and higher resistance to mechanical stress that can damage the shell [36].

Binder solutions (2% *w*/*w*) of sodium alginate, chitosan, or hydroxypropyl methylcellulose (HPMC) were prepared by dispersing polymers into water, or into acetic solution in the case of chitosan. Commonly used concentrations of chitosan and sodium alginate for pellet formulation found in papers vary from 2% to 16% [37]. Dvořáčková K. et al. [38] prepared pellets containing theophylline, microcrystalline cellulose, and chitosan by two different methods. One batch was prepared with chitosan (1–5%) in the state of binder acetic solution, and this was compared with pellets containing 25% chitosan, prepared by wetting with acetic acid. They found that it was necessary to use chitosan in the form of a solution for prolonged liberation of the drug. Hypromellose (HPMC), particularly Methocel K100M was used as a reference polymer for physicochemical property comparison and dissolution behavior comparison with the pellets containing natural polymers chitosan and sodium alginate. HPMC is a swelling polymer with well-established properties and mechanisms of drug release. The mechanism of drug release from the matrix containing HPMC depends on the drug solubility. Soluble drugs are released by diffusion through the gel layer of the polymer and slightly soluble or insoluble drugs are released by the mechanism of matrix erosion. An HPMC molecule is based on a linear polysaccharide cellulose chain with ether-linked methoxyl and hydroxypropyl side groups. The types of HPMC (represented by letters K, E, and F) differ by their substitution degrees and ratios of these side groups, resulting in different abilities to swell and form a gel layer and in a different rates of hydration of the polymer. The methocel type K used in our study possesses the fastest rate of hydration [22,35]. 

Pellets were prepared by the extrusion–spheronization process, where a die of 0.8 mm diameter was used. In the pharmaceutical industry, pellets of medium particle sizes from 600 to 2000 μm are used for most applications. Pellet size can also be classified as conventional (700–1200 µm) and mini (380–550 µm) [30]. The pellet size is important for the subsequent technological operations, specifically filling into hard gelatin capsules or compressing into MUPS tablets, but also for the fate of the dosage form in the gastrointestinal tract. Particles less than 2–3 mm rapidly pass the pylorus, regardless of the filling level of the stomach or the size and density of chyme [13]. The ratio of liquid to powder material and the size of the extruder die significantly influences the quality of the extrudates. The final drying ensures pellet hardness. For this reason, the drying process was maintained till the pellet’s moisture was less than 3%.

### 3.2. Characterisation of Pellets

#### 3.2.1. Measurement of Pellet Size, Shape, and Sphericity

**Figure 1:**
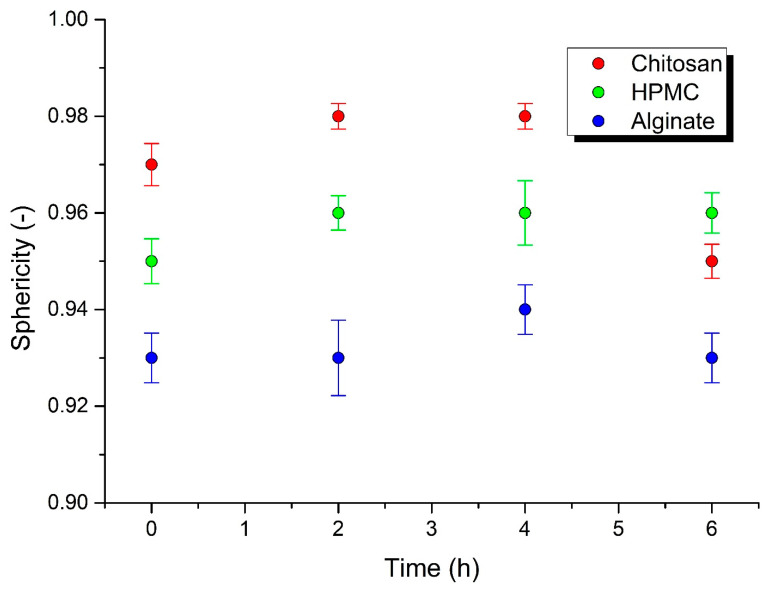
Sphericity change during dissolution testing.

**Figure 2:**
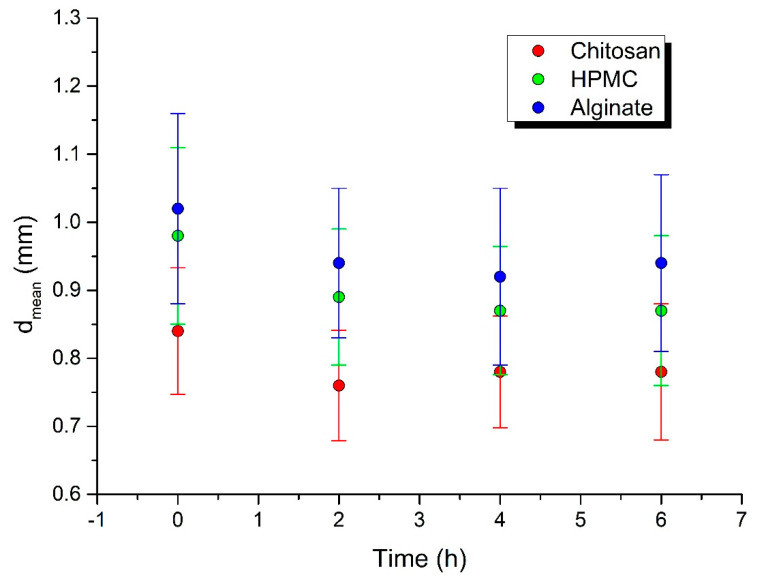
Mean pellet size change during dissolution testing.

A spherical shape is a significant advantage for the good flowability important for processes such as filling into capsules and dies, and it also influences the flow of pellets during coating. The surface area affects the drug release and results in batch-to-batch variability. To ensure the production of consistent shape pellets, the surface area is analyzed by particle size distribution. Particle size distribution parameters: d10, d50, and d90 diameters correspond to 10%, 50%, and 90% of the particle size distribution. DIA data on particle shape, size, and distribution can be used to predict the flow and packing behavior of granular materials. Some authors have investigated various morphological parameters for the characterization of particle shape, using the DIA method on a PartAn 3D device, such as the shape parameters sphericity, aspect ratio, roundness, and concavity [25]. On the basis of sphericity, Maroof et al. [39] classified particles with φ = 0.45 to 0.6 as medium sphericity, φ = 0.6 to 0.8 as spherical, and φ = 0.8 to 1.0 as high sphericity. According to this categorization, all the pellets prepared in this work possessed high sphericity. The sphericity value φ decreased in the following order: chitosan φ = 0.97 > HPMC φ = 0.95 > sodium alginate φ = 0.93 (Table 1). We measured the mean pellet size and sphericity before the dissolution testing and during the dissolution testing of the pellets, which were withdrawn from the dissolution apparatus after 2, 4, and 6 h. The sphericity was maintained during the entirety of the dissolution testing in all of the batches (Table 2, Figure 1). The biggest mean pellet size change was observed after 2 h of dissolution testing, later it remained basically the same (Table 2, Figure 2), which was in accordance with the drug release and matrix erosion evaluation, as discussed later.

#### 3.2.2. Surface Morphology

**Figure 3:**
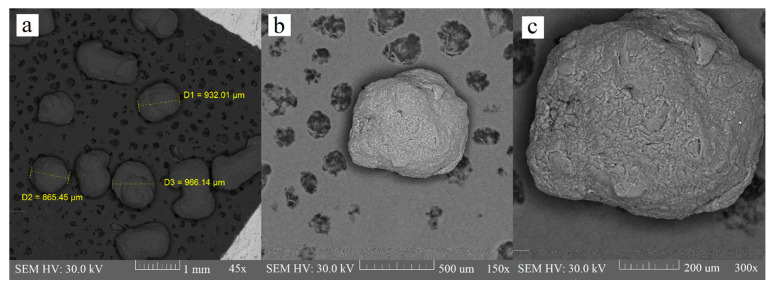
Alginate pellets at 45× (**a**), 150× (**b**), and 300× (**c**) magnification.

**Figure 4:**
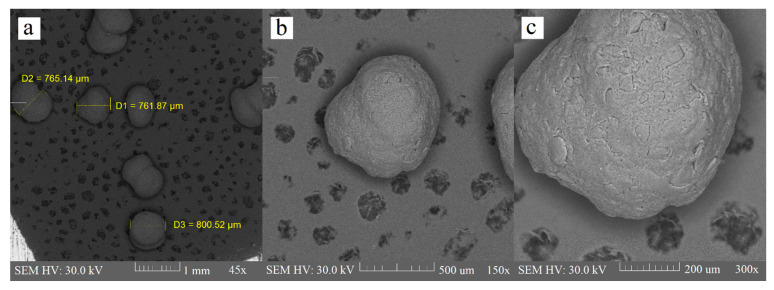
HPMC pellets at 45× (**a**), 150× (**b**), and 300× (**c**) magnification.

**Figure 5:**
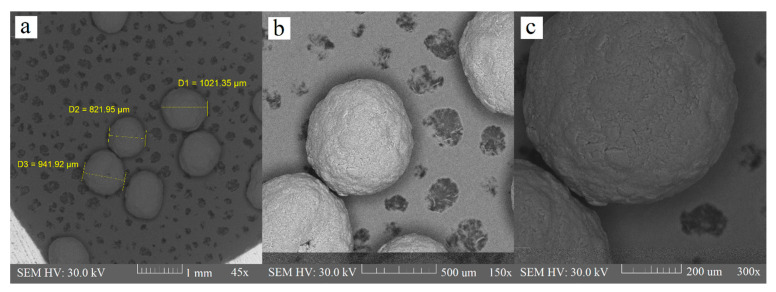
Chitosan pellets at 45× (**a**), 150× (**b**), and 300× (**c**) magnification.

Extrusion is the critical phase of the extrusion–spheronization method because it influences the spheronization and quality of the final product; namely, the mechanical properties, deformability, and plasticity of a wet blend, which is extruded through a shaping die are responsible for the pellet shape. Extrudate, which is broken into rods during the spheronization process, will not produce a spherical product if it does not exhibit sufficient plasticity [35]. Some researchers [30] also used polyvinylpyrrolidone (PVP) as a part of the binder liquid for chitosan pellets, to improve the consistency of wet mass, but it was proven that this is not necessary when using an acidic environment for chitosan dispersion. There were minimum elongated particles in the chitosan batch in comparison with the sodium alginate and HPMC batch. Contrarily, we observed many rods and dumbbells in the sodium alginate batch. HPMC batch contained few rods and they were also considerably shorter than the rods in the sodium alginate batch (Figure 3, Figure 4 and Figure 5). Particle shape is described in three major forms: sphericity and roundness, and also roughness. Some researchers have suggested using charts for visual comparison and description of the particle shape. These charts could facilitate the estimation of particle roundness and sphericity using visual comparisons, which present reference particle silhouettes [39]. Even if a ball-shaped extrudate is imperfect, the evenness of the surface still creates good conditions for possible subsequent coating. The sodium alginate batch was very rough and bumpy, and we also observed a few passageways. The HPMC and chitosan pellets were relatively rough, and less bumpy, as evaluated by the charts mentioned by Maroof et al. (Table 1, Figure 3, Figure 4 and Figure 5).

#### 3.2.3. Particle Size Distribution Estimation by Analytical Sieving

Sieve analysis is the most simple and economical method used to determine pellet size and their distribution. Mechanical sieving is most suitable when most of the particles are larger than about 75 μm. The method is essentially a two-dimensional estimate of size (PhEur 10.6). Using sieve analysis, the pellets were sorted according to their size into seven size fractions. The percentage of each fraction is recorded in Table 3. It shows that the highest proportion was represented by the size fraction 710–900 μm, which means 64.51% in the case of CHIT pellets, 50.62% in the case of ALG pellets, and 75.42% in the case of HPMC pellets. Since a grid with an inner hole size of 0.8 mm was used in the extruder, it was expected that the size fraction 710–900 μm would have the highest proportion. This size is optimal for filling pellets into capsules, as well as for MUPS tablet pressing. Therefore, this size fraction was used for the subsequent tests (dissolution) and MUPS tablet preparation, as well as further characterization. The results of a sieve analysis are important for further technological processes because it is possible to predict which type of pellet formulation would have the smallest “waste” (particles smaller than 250 μm) and to select a composition with the highest production efficiency, i.e., with the smallest material loss.

#### 3.2.4. Mechanical Resistance of Pellets

Pellets possessing the required mechanical resistance are able to withstand the subsequent high attrition during coating, filling into hard gelatin capsules, and compressing. The aim of the mechanical resistance evaluation was to measure the weight loss of the pellets after mechanical agitation. Chitosan pellets revealed a 0% weight loss after the agitation, which means they were 100% mechanically resistant. The HPMC and alginate pellets also showed perfect mechanical properties, because the weight loss after the agitation did not exceed more than 0.15% in any of the batches (Table 4).

#### 3.2.5. Determination of Matrix Erosion

The matrix erosion, expressed as the difference between the initial and the final weight of the pellets during the dissolution testing, corresponded with the drug release. Acyclovir was released simultaneously with the matrix being eroded. Thus, an extensive drug release was observed in the first hour. The matrix erosion of chitosan, sodium alginate, and HPMC pellets reached their limits after 2 h of dissolution, and it can be concluded that the matrix erosion values of chitosan pellets (36.23%) and sodium alginate pellets (36.83%) were equal to matrix erosion value of HPMC pellets (36.75%) (Table 5).

### 3.3. Resistance to Crushing of MUPS Tablets

A tablet requires a certain amount of strength to withstand the mechanical stress of handling in its manufacture, packing, and shipping. To compare the pressability of individual pellets into MUPS tablets, the same pressing conditions (i.e., pressing pressure, set volume of the matrix) had to be maintained during compressing. The effect of the binders on the physical properties of the pellets was not significant. Conversely, the quality of the compressed MUPS tablets showed the influence of the binders, especially during the resistance to crushing test. The strength of the MUPS tablets increased in the following order: CHIT < HPMC < ALG. This knowledge is consistent with the results of the study [40] evaluating the effect of binders on the properties of tablets, where a sharp increase in strength was observed with sodium alginate as a binder, while HPMC and HPC did not have a significant effect on the strength of the tablets (Table 6).

### 3.4. Dissolution Profiles

The formation of dissolution profiles is an essential part of the investigation of the oral solid dosage forms, allowing characterizing or predicting the mechanism and rate of the drug release from the pharmaceutical dosage form. It is necessary to ensure that drug dissolution occurs in an appropriate manner. The dissolution profile shows the amount of drug released from the dosage form into the dissolution media per unit of time. Many factors influence the drug release, such as drug solubility, dose, molecular weight, particle size, shape, physical state, diffusion in polymer, dissolution media, etc. [41].

**Figure 6:**
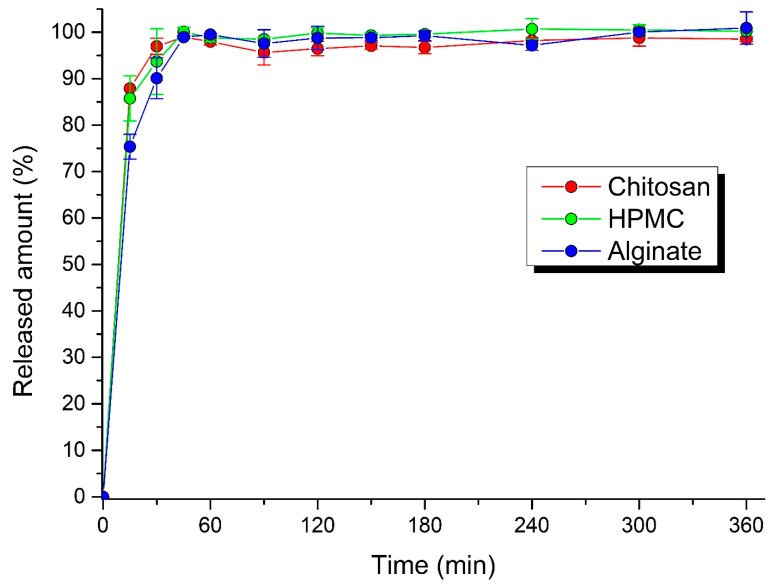
Dissolution profiles of pellets filled in hard gelatin capsules in gastric fluid.

**Figure 7:**
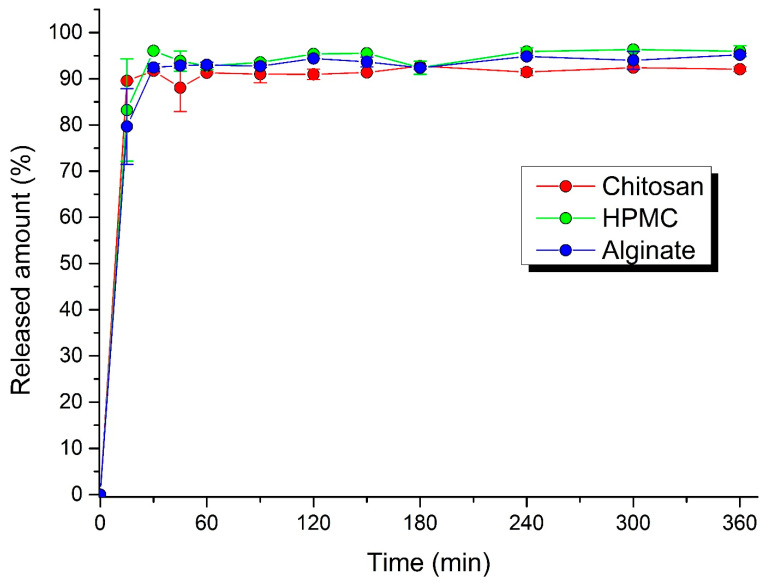
Dissolution profiles of MUPS tablets in gastric fluid.

The process of acyclovir dissolution from the pellets and MUPS tablets is shown in Figure 6 and Figure 7. The dissolution profiles retained in all cases a conventional drug release, with the sudden release of a larger amount of the drug in the first minutes. In the first 15 min, 75.4 ± 2.7% of drug was released from alginate pellets, 85.8 ± 4.9% from HPMC pellets, and 87.9 ± 0.1% from chitosan pellets, and 79.7 ± 2.2% from alginate MUPS tablets, 83.2 ± 1.1% from HPMC MUPS tablets, and 89.6 ± 0.1% from chitosan MUPS tablets. This phenomenon is in line with the results of the matrix erosion test, where about a 30% loss in pellet weight was found, while at subsequent time intervals (after 4 and 6 h) the loss was not further increased. The dissolution profiles also manifest that, within 6 h, the total amount of the active substance contained in the pellets and MUPS tablets was released. Dvořáčková et al. [38] stated that in the acidic environment of artificial gastric juice, chitosan dissolves too quickly, so that the gel layer is not able to form, and therefore the drug is released rapidly. A slight deceleration in the first 15 min was observed using sodium alginate as a binder.

Chatchawalsaisin et al. [37] studied the influence of the incorporation of two oppositely charged hydrophilic natural polymers, chitosan and sodium alginate, alone and in combination, on the ability of formulations containing a model drug (paracetamol) to form spherical pellets by the process of extrusion–spheronization and on the properties of the pellet. There was no significant advantage to be gained from using a mixture of the two polymers, in terms of retarding the drug release.

Dynamic image analysis showed that, during the dissolution studies, the pellets were only reduced in size, while maintaining a constant surface geometry and high sphericity. The disintegration of pellets was not observed during the entire dissolution period. Generally, the mechanism of drug release from pellets can occur in three ways: by diffusion, polymer erosion, or eventually by osmosis [42]. Osmotically driven drug release can be achieved by the addition of osmotically active ingredients (e.g., ionic-sodium and potassium chloride; nonionic-sucrose) into the pellet core [43]. In allowing water to enter under the right circumstances, an osmotic pressure can be built up within the interior of the particle. The drug is forced out of the particle into the exterior through the coating. Drug release by erosion is ensured by coatings that are designed to erode gradually with time, thus releasing the drug contained within the particle sequentially. The most common mechanism of drug release is diffusion, when, due to contact with aqueous fluid in the gastrointestinal tract, water diffuses into the interior of the particle. Drug dissolution occurs and the drug solutions diffuse across the release coating to the exterior. Diffusion through aqueous pores occurs when a continuous, but inhomogeneous, coating layer is punctured with pores. This mechanism is more likely to be operative for coatings formed from aqueous dispersions and when the pellets come into contact with an aqueous medium [44].

### 3.5. Similarity of Dissolution Profiles

The *f*_1_ value was equal to zero when the test and reference profiles were identical, and it increased as the two profiles became less similar. The *f*_2_ value was equal to 100 when the test and reference profiles were identical, and exponentially decreased as the two profiles became less similar. The *f*_1_ values up to 15 (0–15) and *f*_2_ values greater than 50 (50–100) suggest the “similarity” of the two dissolution profiles [34]. Given the data in Table 7, it can be stated that the similarity was not only between the dissolution profiles of drug from the pellets compared to the HPMC pellets used as the reference, but also between the dissolution profiles of the drug released from pellets versus MUPS tablets (e.g., alginate pellets versus alginate MUPS tablet, etc.).

## 4. Conclusions

Pellets, as a microparticulate delivery system, provide significant benefits for oral administration. Compared to oral granules, pellets have a regular spherical shape and, due to more complex technological manufacturing procedures, they are also more mechanically resistant. These facts were confirmed by the excellent results of the sphericity test and the mechanical resistance, while the highest sphericity was achieved under the influence of the chitosan binder. The prepared pellets, with drug release influenced by matrix erosion, were mechanically resistant (near 100%), no matter which binder was used. In contrast, the mechanical resistance of the MUPS tablets, pressed under the same conditions, altered significantly, depending on the binder used in the pellets, while the lowest was recorded with chitosan. Another important finding is that the dissolution profiles, depicting the release of acyclovir under in vitro conditions from pellets filled into capsules and MUPS tablets, were remarkably similar, as evidenced by the similarity factor f2 moving from 61.19 to 65.41. Due to the detection process, it can be concluded that pressing pellets will not cause their destruction. To slow down the drug release, either the technique of coating the pellets themselves, or directly coating MUPS tablets in polymers that slowly dissolve in gastric and intestinal fluid, have to be used for this purpose. Finally, it can be stated that chitosan and alginate sodium are suitable natural binder alternatives to HPMC, ensuring a similar quality, physical properties, and drug release from pellets.

## Figures and Tables

**Table 1:** Measurement of pellet sphericity.

	Measured Value	Particle Shape Description	
	Sphericity–φ	Sphericity	Roughness
**CHIT**	0.97	High sphericity	Relatively rough
**HPMC**	0.95	High sphericity	Relatively rough
**ALG**	0.93	High sphericity	Very rough

**Table 2:** Pellet size alteration and sphericity alteration during dissolution testing.

CHIT Pellets						
Time (h)	d10	d50	d90	Sphericity	Dmean	std
0	0.75	0.83	0.93	0.97	0.84	0.093
2	0.69	0.75	0.83	0.98	0.76	0.081
4	0.69	0.76	0.84	0.98	0.78	0.082
6	0.69	0.77	0.88	0.95	0.78	0.1
**HPMC Pellets**						
	**d10**	**d50**	**d90**	**Sphericity**	**Dmean**	**std**
0	0.84	0.98	1.11	0.95	0.98	0.13
2	0.76	0.89	0.99	0.96	0.89	0.1
4	0.76	0.86	0.98	0.96	0.87	0.094
6	0.75	0.86	0.98	0.96	0.87	0.11
**ALG Pellets**						
	**d10**	**d50**	**d90**	**Sphericity**	**Dmean**	**std**
0	0.87	1	1.18	0.93	1.02	0.14
2	0.81	0.93	1.05	0.93	0.94	0.11
4	0.79	0.9	1.04	0.94	0.92	0.13
6	0.81	0.92	1.05	0.93	0.94	0.13

**Table 3:** Particle size distribution estimation of the pellets containing chitosan (CHIT), sodium alginate (ALG), or hydroxypropyl methylcellulose (HPMC) as a binder.

	CHIT	ALG	HPMC
Aperture Diameter of the Sieve (μm)	Remainder at the Sieve (%)	Remainder at the Sieve (%)	Remainder at the Sieve (%)
1250	0.06	18.42	0.15
900	0.23	28.63	20.87
710	64.51 *	50.62 *	75.42 *
500	23.32	2.20	3.35
355	4.22	0.01	0.02
250	3.88	0.00	0.00
<250	3.68	0.11	0.04

* the most represented size fraction.

**Table 4:** Mechanical resistance of the pellets containing chitosan (CHIT), sodium alginate (ALG), or hydroxypropyl methylcellulose (HPMC) as a binder.

	Weight of the Sample (g)	Rest of the Pellets after Shaking (g)	Mechanical Resistance (%)	Mean Mechanical Resistance (%)
**CHIT**	15.00	15.00	100.00	
	15.00	15.00	100.00	**100.00**
	15.00	15.00	100.00	
**ALG**	15.00	15.00	100.00	
	15.00	14.99	99.93	**99.98**
	15.04	15.04	100.00	
**HPMC**	15.01	14.99	99.87	
	15.00	15.00	100.00	**99.93**
	15.00	14.99	99.93	

**Table 5:** Determination of the matrix erosion of chitosan (CHIT), hydroxypropyl methylcellulose (HPMC), and sodium alginate (ALG) pellets.

Pellet Type	Weigh of the Sample (g)	Matrix Erosion (%)
**CHIT**/before dissolution	0.400	**-**
**CHIT**/2 h dissolution	0.255	**36.23**
**CHIT**/4 h dissolution	0.257	**35.65**
**CHIT**/6 h dissolution	0.255	**36.28**
**HPMC**/before dissolution	0.400	**-**
**HPMC**/2 h dissolution	0.253	**36.75**
**HPMC**/4 h dissolution	0.253	**36.78**
**HPMC**/6 h dissolution	0.253	**36.83**
**ALG**/before dissolution	0.400	**-**
**ALG**/2 h dissolution	0.253	**36.83**
**ALG**/4 h dissolution	0.254	**36.45**
**ALG**/6 h dissolution	0.250	**37.43**

**Table 6:** Resistance to crushing of the MUPS tablets produced from pellets containing sodium alginate (ALG), chitosan (CHIT), or hydroxypropyl methylcellulose (HPMC) as a binder.

Sample	ALG	CHIT	HPMC
	Force (N)	Force (N)	Force (N)
1.	118	40	90
2.	122	40	105
3.	96	45	105
4.	110	45	78
5.	109	49	95
6.	96	39	98
7.	87	48	96
8.	104	36	66
9.	98	39	70
10.	100	39	110
**Average**	104	42	91.3
**S.D.**	10.80	4.40	15.19

**Table 7:** The difference factor (*f*_1_) and the similarity factor (*f*_2_) comparing the reference (*R*) versus the tested (*T*) formulation (*R*/*T*).

	Pellets		MUPS		Pellets/MUPS		
	HPMC/ALG	HPMC/CHIT	HPMC/ALG	HPMC/CHIT	ALG	CHIT	HPMC
*f*_1_	2.20	1.32	1.73	3.45	4.49	6.98	4.79
*f*_2_	71.70	79.44	83.54	68.59	63.96	60.19	65.41

## Data Availability

Not applicable.
